# Glioma Initiating Cells Form a Differentiation Niche Via the Induction of Extracellular Matrices and Integrin αV

**DOI:** 10.1371/journal.pone.0059558

**Published:** 2013-05-21

**Authors:** Akiko Niibori-Nambu, Uichi Midorikawa, Souhei Mizuguchi, Takuichiro Hide, Minako Nagai, Yoshihiro Komohara, Megumi Nagayama, Mio Hirayama, Daiki Kobayashi, Nobuyuki Tsubota, Tatsuya Takezaki, Keishi Makino, Hideo Nakamura, Motohiro Takeya, Junichi Kuratsu, Norie Araki

**Affiliations:** 1 Department of Tumor Genetics and Biology, Graduate School of Medical Sciences, Kumamoto University, Kumamoto-city, Japan; 2 Healthcare Systems Laboratories, Sharp Corp. Chiba-city, Japan; 3 Department of Neurosurgery, Graduate School of Medical Sciences, Kumamoto University, Kumamoto-city, Japan; 4 Department of Cell Pathology, Graduate School of Medical Sciences, Kumamoto University, Kumamoto-city, Japan; University of Michigan School of Medicine, United States of America

## Abstract

Glioma initiating cells (GICs) are considered responsible for the therapeutic resistance and recurrence of malignant glioma. To clarify the molecular mechanism of GIC maintenance/differentiation, we established GIC clones having the potential to differentiate into malignant gliomas, and subjected to DNA microarray/iTRAQ based integrated proteomics. 21,857 mRNAs and 8,471 proteins were identified and integrated into a gene/protein expression analysis chart. Gene Ontology analysis revealed that the expression of cell adhesion molecules, including integrin subfamilies, such as α2 and αV, and extracellular matrices (ECMs), such as collagen IV (COL4), laminin α2 (LAMA2), and fibronectin 1 (FN), was significantly upregulated during serum-induced GIC differentiation. This differentiation process, accompanied by the upregulation of MAPK as well as glioma specific proteins in GICs, was dramatically accelerated in these ECM (especially FN)-coated dishes. Integrin αV blocking antibody and RGD peptide significantly suppressed early events in GIC differentiation, suggesting that the coupling of ECMs to integrin αV is necessary for GIC differentiation. In addition, the expression of integrin αV and its strong ligand FN was prominently increased in glioblastomas developed from mouse intracranial GIC xenografts. Interestingly, during the initial phase of GIC differentiation, the RGD treatment significantly inhibited GIC proliferation and raised their sensitivity against anti-cancer drug temozolomide (TMZ). We also found that combination treatments of TMZ and RGD inhibit glioma progression and lead the longer survival of mouse intracranial GIC xenograft model. These results indicate that GICs induce/secrete ECMs to develop microenvironments with serum factors, namely differentiation niches that further stimulate GIC differentiation and proliferation via the integrin recognition motif RGD. A combination of RGD treatment with TMZ could have the higher inhibitory potential against the glioma recurrence that may be regulated by the GICs in the differentiation niche. This study provides a new perspective for developing therapeutic strategies against the early onset of GIC-associated glioma.

## Introduction

Malignant glioma is the most common and lethal primary brain tumor [1]. Recently, it was proposed that glioma development is initiated and maintained by glioma initiating cells (GICs), a population of cells capable of extensive self-renewal, multi-lineage differentiation, and promotion of glioblastoma multiform (GBM) development, in immunodeficient mice [2]. It is suggested that GICs are responsible for the therapeutic resistance and recurrence of GBM [3], and thus, considered the most effective therapeutic target for the treatment of malignant gliomas.

It is suggested that GICs reside in a microenvironment referred to as the niche, which is composed of stem cells, neighboring supportive cells, extracellular matrix (ECM), and other factors required for stem cell renewal [4], and the manipulation of GIC maintenance/differentiation could be applicable for the clinical treatment of malignant glioma. Previous studies showed that several signaling pathways, regulate GIC maintenance [5], [6], however, the molecular mechanism or the factors controlling GIC differentiation have not been clearly identified.

To understand the regulation mechanism of GICs, both transcriptome and proteome analysis of global changes in GICs related to the differentiation/maintenance are the most powerful strategies; however, these analyses, especially proteomics, have not been used intensively in this field. We previously established a concise proteomic strategy consisting of sequential MS-based, *in silico*, and cell biological analyses to study the mechanisms of neural differentiation using neural stem cell (NSC) models [7], and demonstrated that this approach was effective for elucidating the functions of proteins involved in cellular biological processes.

In this study, we established GIC clones from tumors of malignant glioma patients and subjected them to integrated proteomics to survey the proteins and mRNAs differentially expressed in GICs after they have been induced by serum stimulation that is generally utilized as a method for differentiation assay of GIC [8], [9]. The results showed that the expression of cell adhesion molecules and ECMs was increased during GIC differentiation and that serum factor-induced coupling of ECMs to integrin αV via the RGD motif are absolutely important for early events in GIC differentiation and proliferation. Additionally, the *in vivo* as well as *in vitro* results raised the possibility that GIC induces/secretes ECMs by itself to form a specific microenvironment, called the “differentiation niche”, which facilitates the development of malignant glioma and could be the most likely candidate for the therapeutic target of GIC-associated glioma recurrences. This study provides new insights into the molecular mechanism of the GIC differentiation via integrins and ECMs on their specific microenvironment, and functional target for the early onset of GIC-associated glioma.

## Results

### Establishment and characterization of tumorigenic GICs from human malignant gliomas

**Figure 1:**
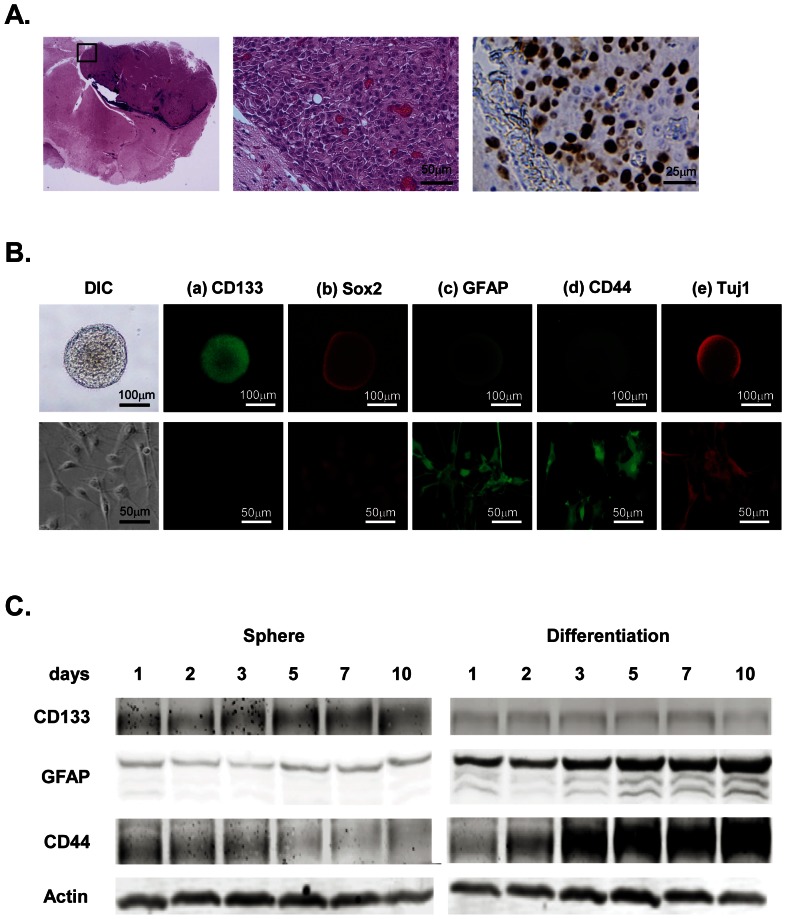
Characterization of GICs established from tumors of malignant glioma patients. ***A***. Histochemical observations of glioblastomas developed from mouse brain GICs xenografts. A representative H&E staining pattern of a glioblastoma derived from a GICs xenograft in NOD/SCID mouse brain (left and center), and immunohistochemistry of a proliferation marker Ki67 (right). ***B***. GIC spheres (a–e, upper photos) or differentiating cells in the presence of 10% FCS (a–e, lower photos) after 7 day's culture in non-coated dishes were immunostained to analyze the expression patterns of the neural marker proteins with specific antibodies as indicated. Secondary antibodies labeled with Alexa 488 (green) and Alexa 546 (red) were used for the detection. ***C***. GIC spheres in the NSC medium or differentiating cells in the presence of 10% FCS after the indicated periods of cultures in non-coated dishes were subjected to the SDS-PAGE followed by the western blotting using anti-CD133, GFAP and CD44 antibodies to compare their expression patterns in both types of GICs. Actin was used for the internal control.

We isolated eight GIC clones from four GBM and one AO tumors. GIC spheres were sub-cloned, and, GIC-03A, -03U, -06A, -06U, -07U, -08U, -09A, and -09U clones were continuously maintained for more than two years. Among all of the clones, GIC07U, 03A and 03U (**Fig. S1A**) had the highest capacity for sphere formation and self-renewal, and the transplantation of these cells into the mouse brain resulted that all of the mice died at 4–16 weeks after the injection. They formed an expanded malignant glioma with a high proliferation index in each xenograft (**Fig. 1A**), suggesting that GIC03A, 03U and 07U possess aggressive tumorigenesity. Using these GIC clones, we established an *in vitro* glioma induction system by using serum stimulation [8], [9]. Upon the stimulation, the GIC spheres showed increased cellular proliferation, motility, filopodia/lameripodia formation and adhesion to the culture dishes, and importantly the NSC marker CD133 and Sox2 expressions were decreased with time dependent manner (**Fig. 1B** **a, b,** **Fig. 1C** **, and Fig. S1B**). Simultaneously, the astrocyte/glioma marker GFAP and the malignancy marker CD44 dramatically expressed upon serum stimulation with higher levels, but those of the neuron marker Tuj1 were not (**Fig. 1B** **c–e,** **Fig. 1C** **, and Fig. S1B**), demonstrating that the GIC clones had both the characteristics of NSCs and the capacity to differentiate into glioma cells, and that they were also capable of long-term self-renewal, differentiation, and tumorigenesis. These phenotypes of the GIC03A, 03U and 07U were consistent with those of the GICs/GSCs (glioma stem cells) that have been established and reported in elsewhere [10].

### Integrated differential analyses of mRNA and proteins in GIC03A/GIC03U under sphere formation and differentiating conditions

**Figure 2:**
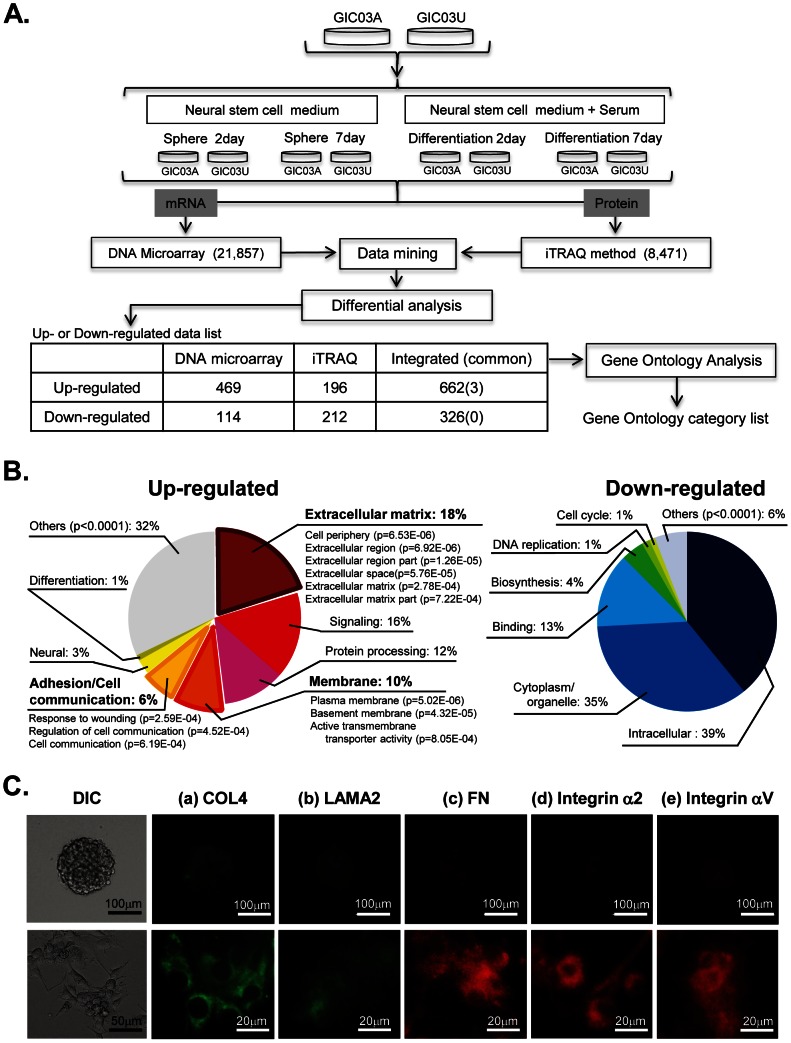
Integrated proteomics, GO analysis and immunocytochemical validation of GICs. ***A***. A workflow for the identification of the genes regulating GIC differentiation. At first, each GIC sphere was disassociated into single cells, separated into four fractions, and cultured in NSC medium containing growth factors or 10% FCS for 2 days or 7 days. Cells were collected and washed, and mRNA and proteins were simultaneously prepared and subjected to transcriptome and proteome analyses, respectively. mRNA differential analysis using DNA expression arrays quantitatively identified 21,857 expressed genes. Proteome differential analysis using the iTRAQ method identified 8,471 proteins from 564,657 peptides. All the data were integrated into one chart, and used for further GO and functional analyses. ***B***. Pie charts of the highly extracted GO terms functionally grouped as upregulated genes/proteins (left) or as downregulated genes/proteins (right) during GIC differentiation (Table S1). The GO term frequencies of each functional group (*p*<0.001) among 2,046 and 1,868 terms in up (662)- and down (326)-regulated genes/proteins, respectively, are shown as percentages. ***C***. Validation of the expression of ECMs and integrin families by immunocytochemistry. GIC spheres (a–e, upper panel) or differentiating cells in the presence of 10% FCS (a–e, lower panel) after 7 day's culture in non-coated dishes were immunostained to analyze the expression patterns of the identified proteins upregulated in differentiating GICs.

To study the dynamic changes at the molecular level in the established GIC clones upon their differentiations, mRNA and protein expression in both GIC03A and GIC03U was analyzed in the presence and absence of serum stimulation using the integrated proteomics procedure (**Fig. 2A**). mRNA differential analysis quantitatively identified 21,857 expressed genes. On the other hand, proteome differential analysis using the iTRAQ method identified 8,471 proteins from 564,657 peptides. All the data were integrated into one chart by an integrated gene/proteomic expression analysis chart application. The upregulated 469 mRNAs and 196 proteins, or the downregulated 114 mRNAs and 212 proteins in GIC03A and -03U cells at 2 and 7 days after serum stimulation were extracted by Subio platform, and subjected to GO analysis. The identified genes/proteins were classified into the following functional groups: upregulated groups, extracellular matrices (ECMs) (18%); signaling (16%); protein processing (12%); membrane (10%); adhesion/cell communication (6%); and downregulated groups; intracellular (39%); cytoplasm/organelle (35%); binding (13%); biosynthesis (4%), and others (**Fig. 2B** **and Table S1**). We also observed the upregulation of glioma specific proteins [11] in the differentiating GIC03A and GIC03U, such as vimentin, VEGF, EGFR, MAPKs, KRAS, Musashi, FABP7, S100B, Cathepsin B, BAX, BAD, BID, CDK4, CDK6, as well as CD44, GFAP in proteomics or mRNA levels during 2 to 7 days of the serum stimulation of GICs (**Table S2**). These results suggest that the serum stimulation of GIC spheres downregulates their stemness properties and induces their differentiation activities to glioma or their progenitor formation.

### Validation of the expression of ECMs and integrin families

Among upregulated functional groups in differentiating GICs, we focused the adhesion molecules, including integrin subfamily proteins, and ECMs, such as collagen (COL) family members, laminin (LAM), and fibronectin (FN) (**Table 1**). To confirm the results obtained by integrated proteomics of GICs, the expression of integrin family proteins, such as integrin α2 and αV, and ECMs, such as COL type IVα1 (COL4A1), LAM α2 (LAMA2), and FN1 which are significant in this study was validated by immunocytochemistry and western blot analysis. The results confirmed that all of these proteins were apparently expressed (although the LAMA2 level was relatively weak) during the differentiation of GIC03A, 03U, and 07U (**Fig. 2C**). This was especially the case for integrin αV and FN on day 7 of the differentiation (**Fig. S2**).

**Table 1:** mRNA and proteins belonging to functional groups with GO terms for ECMs, membrane proteins, and adhesion/cell communication.

Entrez Gene ID	Gene symbol	Full name	Fold change ratio							
			mRNA				Protein			
			2day		7day		2day		7day	
			Average ratio	S.E.	Average ratio	S.E.	Average ratio	S.E.	Average ratio	S.E.
960	*CD44*	CD44 molecule	6.97	3.31	1.16	0.28	1.55	0.43	3.15	0.92
1301	*COL11A1*	collagen, type XI, alpha 1	6.82	2.63	0.89	0.27	0.84	0.07	1.20	0.11
1290	*COL5A2*	collagen, type V, alpha 2	3.20	0.69	1.02	0.20	ND	ND	ND	ND
3912	*LAMB1*	laminin, beta 1	2.57	0.42	1.67	0.10	0.91	0.05	1.78	0.34
3689	*ITGB2*	integrin, beta 2	2.39	1.53	1.91	0.69	0.56	0.08	0.42	0.14
1292	*COL6A2*	collagen, type VI, alpha 2	2.28	0.08	6.55	4.83	ND	ND	ND	ND
3676	*ITGA4*	integrin, alpha 4 (antigen CD49D)	2.23	0.38	1.59	0.46	ND	ND	ND	ND
2335	***FN1***	**fibronectin 1**	2.21	0.10	1.44	0.15	ND	ND	ND	ND
3914	*LAMB3*	laminin, beta 3	2.07	0.35	1.00	0.16	0.99	0.11	1.13	0.00
3678	*ITGA5*	integrin, alpha 5	1.92	0.53	1.50	0.30	0.87	0.06	1.38	0.23
1299	*COL9A3*	collagen, type IX, alpha 3	1.91	0.93	0.85	0.09	3.01	1.09	2.93	1.03
1282	***COL4A1***	**collagen, type IV, alpha 1**	1.91	0.04	2.23	0.68	4.10	2.87	1.79	0.32
80781	*COL18A1*	collagen, type XVIII, alpha 1	1.84	0.56	1.50	0.03	ND	ND	ND	ND
3675	*ITGA3*	integrin, alpha 3 (antigen CD49C)	1.77	0.12	1.83	0.36	ND	ND	ND	ND
1289	*COL5A1*	collagen, type V, alpha 1	1.71	0.01	1.91	0.80	ND	ND	ND	ND
3674	*ITGA2B*	integrin, alpha 2b (antigen CD41)	1.67	1.03	2.19	0.58	ND	ND	ND	ND
3908	***LAMA2***	**laminin, alpha 2**	1.64	0.84	0.86	0.34	2.56	0.43	2.85	0.89
3911	*LAMA5*	laminin, alpha 5	1.37	0.30	1.67	0.64	0.68	0.10	0.90	0.10
1277	*COL1A1*	collagen, type I, alpha 1	1.35	0.56	1.55	0.34	1.08	0.12	1.19	0.19
1291	*COL6A1*	collagen, type VI, alpha 1	1.14	0.16	1.22	0.16	1.18	0.17	1.33	0.02
169044	*COL22A1*	collagen, type XXII, alpha 1	1.03	0.37	1.17	0.22	6.17	2.54	4.82	4.47
3685	***ITGAV***	**integrin, alpha V (antigen CD51)**	1.02	0.28	0.72	0.01	1.15	0.06	1.58	0.14
3696	*ITGB8*	integrin, beta 8	0.92	0.25	0.80	0.07	0.97	0.08	1.33	0.08
91522	*COL23A1*	collagen, type XXIII, alpha 1	0.90	0.44	1.86	1.07	0.77	0.15	1.39	0.68
3688	*ITGB1*	integrin, beta 1 (antigen CD29)	0.87	0.04	1.06	0.08	0.79	0.09	0.91	0.14
3913	*LAMB2*	laminin, beta 2 (laminin S)	0.86	0.07	0.91	0.32	1.02	0.15	0.88	0.15
3695	*ITGB7*	integrin, beta 7	0.85	0.33	0.38	0.07	ND	ND	ND	ND
81578	*COL21A1*	collagen, type XXI, alpha 1	0.81	0.06	4.72	0.96	ND	ND	ND	ND
1300	*COL10A1*	collagen, type X, alpha 1	0.77	0.46	0.46	0.14	ND	ND	ND	ND
3915	*LAMC1*	laminin, gamma 1	0.65	0.04	0.96	0.11	0.65	0.10	1.14	0.08
1280	*COL2A1*	collagen, type II, alpha 1	0.50	0.06	0.79	0.45	0.85	0.13	0.91	0.04
1306	*COL15A1*	collagen, type XV, alpha 1	0.49	0.46	18.58	4.09	0.75	0.20	0.79	0.02
1297	*COL9A1*	collagen, type IX, alpha 1	0.43	0.04	1.03	0.07	1.01	0.16	0.98	0.10
3673	***ITGA2***	**integrin, alpha 2 (CD49B)**	0.36	0.09	0.82	0.10	1.83	0.29	1.37	0.25
3655	*ITGA6*	integrin, alpha 6	0.35	0.10	1.75	0.18	0.69	0.09	0.58	0.04
3910	*LAMA4*	laminin, alpha 4	0.29	0.16	0.92	0.06	ND	ND	ND	ND
8515	*ITGA10*	integrin, alpha 10	0.21	0.03	0.64	0.30	ND	ND	ND	ND
131873	*COL6A6*	collagen, type VI, alpha 6	0.16	0.11	2.76	2.68	4.17	3.01	6.75	5.16
57642	*COL20A1*	collagen, type XX, alpha 1	0.12	0.01	0.35	0.21	1.31	0.04	3.52	1.73

These genes were up- or downregulated during GIC differentiation at 2 days and 7 days after serum stimulation. The data were obtained from integrated proteomics using integrated protein expression analysis chart. The average ratios calculated from each normalized data (n = 6, in proteomics, n = 2, in DNA microarray) are shown. DN, not detected.

### ECMs accelerate the differentiation of GICs

**Figure 3:**
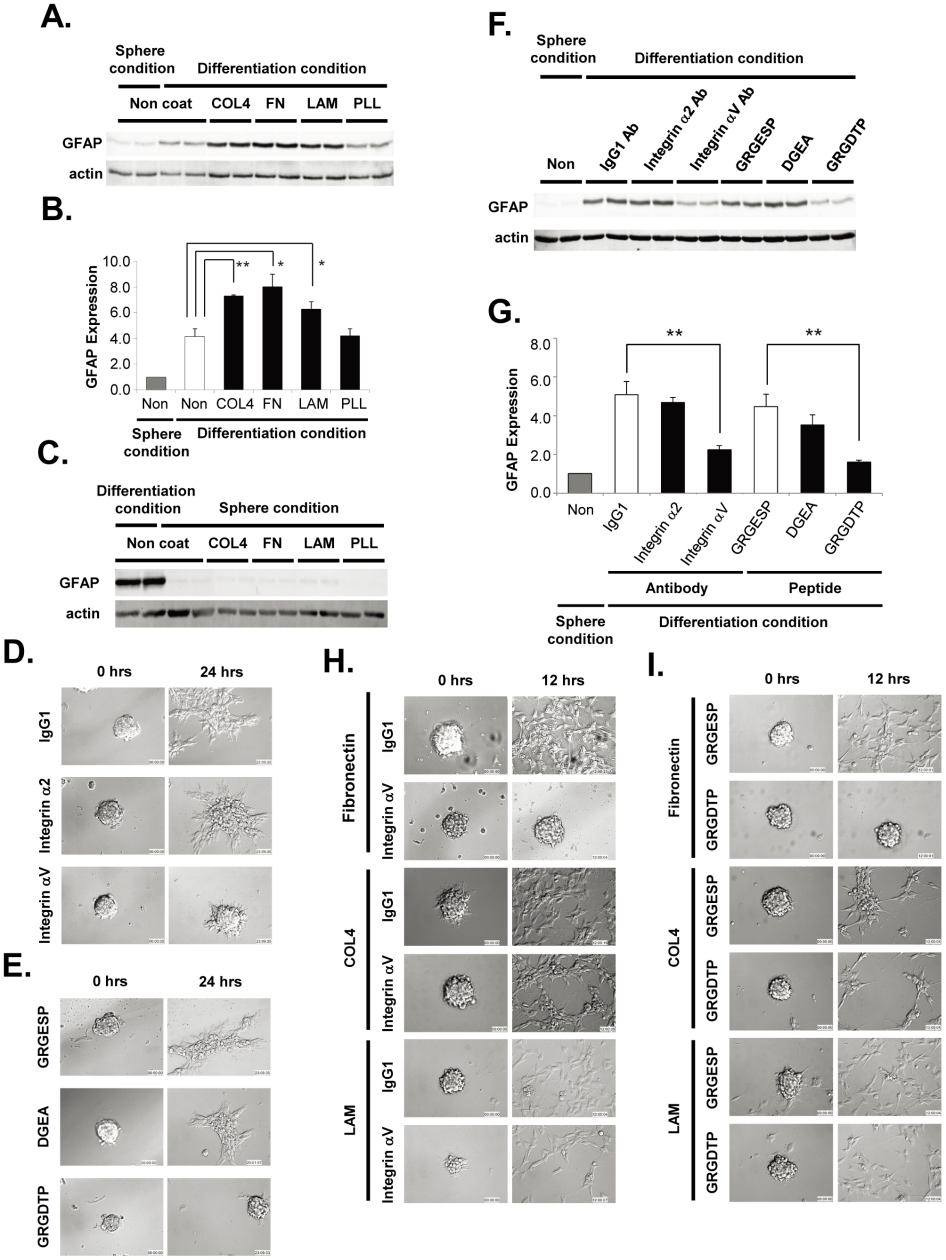
Adhesion, migration and differentiation of GICs on ECMs via integrin αV. ***A***. GFAP expressions in GICs cultured on ECM-coated dishes in the presence/absence of FCS for 48 hours was analyzed by western blotting. ***B***. The intensity ratio of GFAP expression analyzed in (A) was quantitated using an actin internal marker. ***C***. GFAP expressions in GICs cultured on ECM-coated dishes in the presence/absence of serum stimulation. The GIC spheres cultured on ECMs (COL4, LAM, FN) or PLL-coated dishes without serum, or on non-coated dishes with/without serum, were analyzed by western blot analysis at 48 hours after seeding. ***D and E***. GIC spheres treated with blocking antibodies against integrin α2 and αV, or with control IgG (D), or with integrin-binding peptide DGEA, GRGDTP, or control peptide GRGESP (E) were stimulated by serum in non-coated dish. The representative stack photographs of the sphere at 0 hour and after 24 hours of FCS stimulation are shown. ***F***. GFAP expression in GICs treated under the same conditions as in (D) and (E) for 48 hours was analyzed by western blotting. ***G***. The intensity-ratio of the GFAP expression of GICs analyzed in (F) was quantitated. The values shown in (C) and (G) are means ±S.E of three independent experiments run in duplicate. *p<0.05 and **p<0.01. ***H***. Effects of integrin blocking antibodies on GICs adhesion/migration on ECM-coated dishes. GIC spheres were treated with blocking antibodies against integrin αV or control IgG for 30 min before seeding onto ECM-coated dishes. Cell morphologies were analyzed by time-lapse microscopy. Representative stack photographs taken at 0 and 12 hours are shown. ***I***. Effects of integrin-binding peptides on GICs adhesion/migration on ECM-coated dishes. GIC spheres were treated with integrin-binding GRGDTP, or control (GRGESP) peptides for 30 min before seeding into ECM-coated dishes. Cell morphologies were analyzed by time-lapse microscopy, and shown by the representative stack photographs at 0 and 12 hours.

To understand the contribution of ECMs, such as COL4, LAM and FN, to GIC differentiation, GIC spheres were seeded onto ECM-coated dishes containing NSC medium supplemented with 10% FCS, and morphological changes and the differentiation marker expression were analyzed. The adhesion/migration of GIC spheres on each ECM were dramatically promoted within a few hours as compared to cells seeded on uncoated dishes with serum. Importantly, the expression of glioma marker GFAP in GICs on COL4-, LAM- and FN-coated dishes was also dramatically increased within 48 hours, whereas that of cells seeded on uncoated or PLL-coated dishes was much lower (**Fig. 3A, B**). On the other hand, under the NCS medium conditions, GFAP upregulation was not obvious in any of the ECM-coated dishes (**Fig. 3C**). These results demonstrate that GIC differentiation induced by serum stimulation is accelerated by ECM proteins.

### Interaction of Integrin αV and FN accelerates the serum induced differentiation of GICs

Cell adhesion to ECMs stimulates cell differentiation primarily by increasing signaling through integrin-ECM interactions. To determine whether GIC differentiation was mediated by integrin α2, αV, or other integrin families, the effects of integrin inhibitors on the GIC differentiation were studied. Integrin α chain-antibodies or integrin-specific binding peptides (containing RGD or DGEA motif) were used to treat GICs before seeding them onto dishes. GIC spheres were seeded onto uncoated dishes and cultured for 48 hours in serum-containing medium in the presence of integrin antibodies against integrin α2, αV, and control IgG, or in the presence of GRGDTP, DGEA, and GRGESP. The morphology of differentiating GIC spheres was monitored and the cellular inductions of the differentiation marker GFAP were analyzed. The adhesion/migration of the GIC spheres was prominently inhibited by the integrin αV antibody and GRGDTP peptide, whereas the effects of integrin α2 antibody or DGEA peptide were slightly effective but not significant (**Fig. 3D, E** **, and movies S1, S2, S3, S4, S5, S6**), suggesting that upregulated cellular integrin αV and ECM interaction induced by the serum stimulation accelerates GIC differentiation. We also analyzed the effects of other blocking antibodies against integrin α5, α6, and β1, which were reported to act as receptors for ECMs via RGD domain; however, these antibodies were not effective (**Fig. S3**). Importantly, the induction of GFAP in differentiating GICs was significantly inhibited by integrin αV antibody and the RGD peptide (**Fig. 3F, G**), suggesting that integrin αV inhibitors can suppress the early event of the differentiation of GICs. To identify which ECMs directly associated to the integrin αV expressed on differentiating GICs, we analyzed the cell adhesion and migration of GICs seeded on the FN, COL4, or LAM-coated dishes with integrin αV inhibitors. The results showed that integrin αV blocking antibody as well as RGD peptide significantly inhibited the GICs adhesion and migration on FN-coated dishes, whereas, no effect was observed on LAM- or COL4-coated dishes (**Fig. 3H, I**). These results suggest that the interaction of FN and integrin αV is the main factor to accelerate the GIC differentiation.

**Figure 4:**
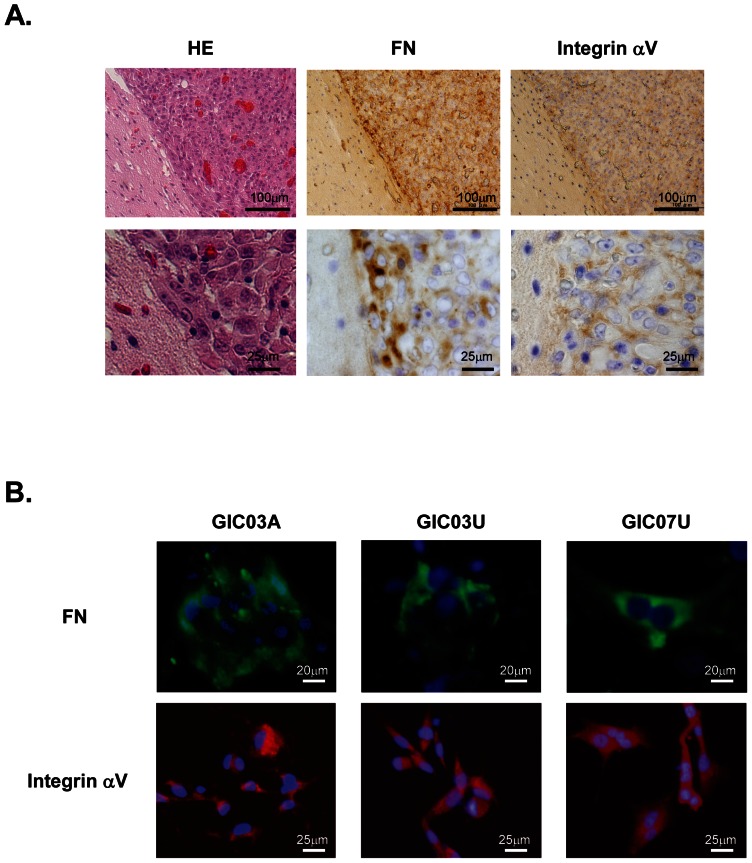
Fibronectin/integrin αV expression in glioblastomas developed from mouse brain GIC xenografts and differentiating GIC clones. ***A***. HE staining and immunohistochemical staining with anti-FN and -integrin αV antibodies of serial sections of a glioblastoma derived from a mouse brain GIC xenograft. The representative tissues were obtained from a glioblastoma in the brain at 39 days after the intracranial injection of GIC07U. ***B***. Differentiating GIC03A, GIC03U and GIC07U were stained with anti-FN antibody (green, upper panel), DAPI (blue; upper panel), anti-integrin αV antibody (red; lower panel) and DAPI (purple; lower panel) after 5 day's serum stimulation. All of GIC clones possess cytoplasmic expression of FN and membrane expression of integrin αV.

Immunohistochemistry confirmed that FN and integrin αV are apparently deposited in GIC-derived glioblastomas that developed from mouse brain xenografts (**Fig. 4A**). Notably, high FN expression in glioma cells migrating to the edge of tumors, and significant expression of integrin αV on those cell membranes were observed (**Fig. 4A** **, lower panel**). Additionally, FN was expressed in the cytoplasmic area of GIC03A, GIC03U and GIC07U differentiating cells, and integrin αV expression on the membrane area of those differentiating cells were also confirmed (**Fig. 4B**). These results demonstrate that FN is obviously secreted from GICs during serum-induced GIC differentiation and the interaction with integrin αV is an important initiation event for upregulation of GIC differentiation and glioma formation.

### Combination treatment of RGD peptide and TMZ suppresses cellular viability of GICs during differentiation in vitro and GIC derived glioma progression in vivo

Finally, we assessed efficacies of RGD peptide in the GIC differentiation and growth *in vitro*, and in the GIC derived glioma propagation *in vivo*.

**Figure 5:**
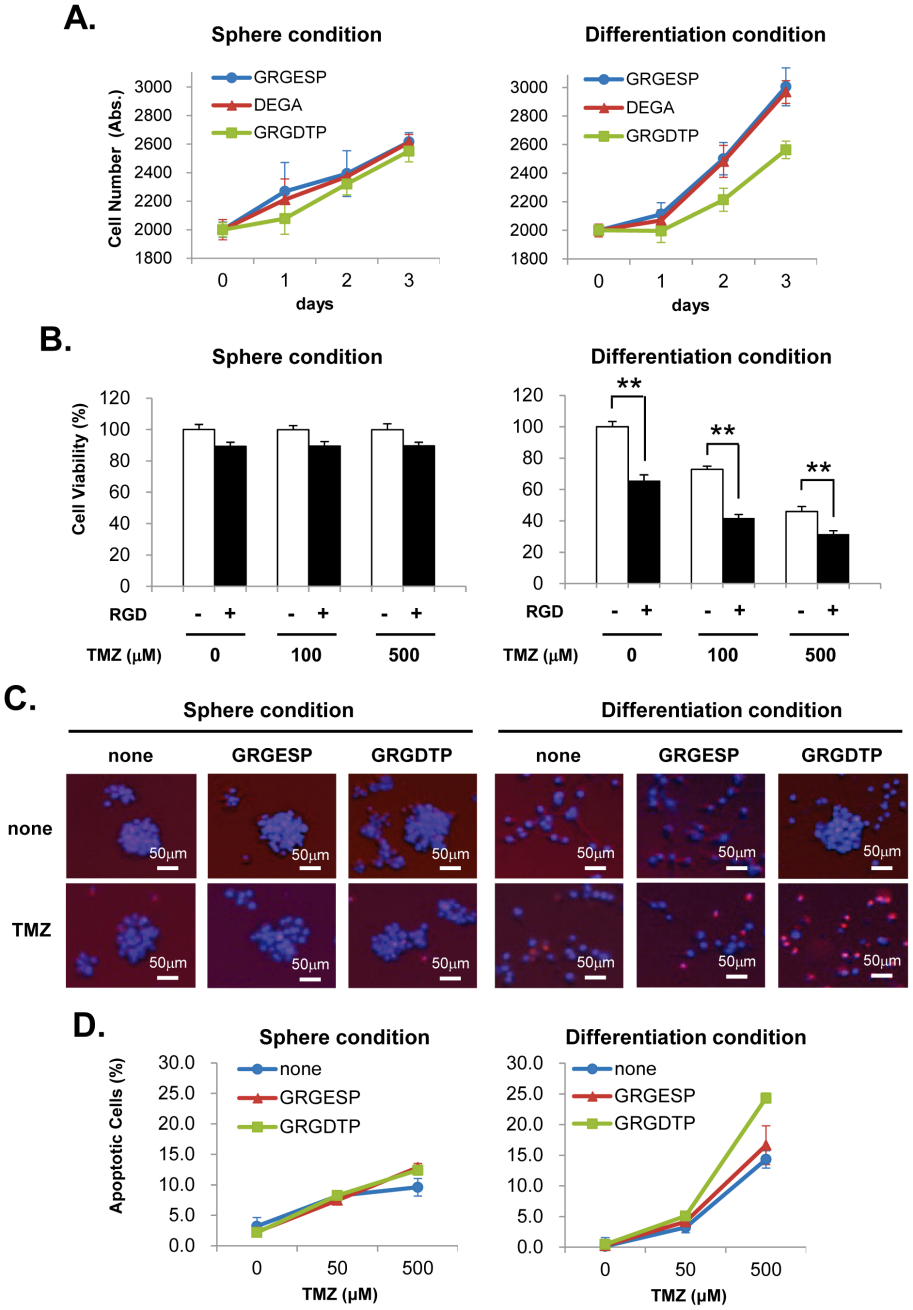
Treatment of RGD peptide and TMZ decreases cellular viability of GICs during serum-induced differentiation. ***A***. GIC spheres or serum induced differentiating GICs were treated with GRGESP as control (blue), and RGDTP (green) or DGEA peptide (red) as integrin-binding peptide for 3 days, and their cellular viabilities were analyzed with WST-8 assay. ***B***. GIC spheres or serum induced differentiating GICs were treated with TMZ (0, 100, 500 µM) and/or RGD peptide for 3 days, and analyzed by WST-8 assay. The values shown in (A) and (B) are means ±S.E of three independent experiments. **p<0.01. ***C***. Representative morphological changes of GIC spheres or differentiating cells after 4 days of the treatments with RGD peptide (300 µM) or TMZ (500 µM) alone, or both of agents. The cells were stained with PI for counting the apoptotic cells (red), and Hoechst 33342 for staining the total cells (blue) ***D***. Effects of the RGD peptide and TMZ on GIC spheres and differentiating cells. GIC spheres or serum induced differentiating cells were treated with an integrin-binding peptide (GRGDTP: green), a control peptide (GRGESP: red), or control buffer (blue), and followed by the treatment of TMZ (0, 100, 500 µM) for 4 days, The percentage of apoptotic cells were calculated from the cell number of PI staining cells (for apoptotic cells) in the Hoechst staining cells (for total cells) by MetaMorph software. Data are shown as the mean of 12-field counts in two experiments ±S.E as described in Material and Methods.

Several synthetic peptides, such as GRGESP, GRGDTP and DGEA, were pretreated with GICs before and after serum stimulation and analyzed their effects for the cellular proliferation. All of the peptides did not show any effect on the cellular proliferation in the GIC spheres (**Fig. 5A** **, left**). On the other hand, the increased proliferation of cells after the serum stimulation was significantly inhibited with RGD peptides to almost same level as those of GIC spheres, however, GRGESP or DGEA peptide did not show any effects (**Fig. 5A** **, right**). These results suggest that RGD peptide effectively inhibits cell proliferation during the GIC differentiation.

The downstream of integrin signals related to the GIC differentiation and proliferation after the serum stimulation was analyzed. As we found the upregulation of MAPK families in the differentiating GICs by integrated proteomics (**Table S2**), we speculated that MAPK signaling pathway could be involved in this system. Firstly, GICs were seeded on the non-coated dish in the presence or absent of serum, and the expression of phosphorylated-MAPK (Phospho-ERK1/2) were analyzed by western blotting. As we expected, the upregulation of phosphorylated-MAPK were significant in the serum stimulated GICs (**Fig. S4**), and upon treatments with RGD peptides, these activations were diminished (**Fig. S5**), suggesting that serum induced differentiation/proliferation of GICs are associated with upregulation of MAPK signals via the integrin αV and FN interaction.

We next analyzed the anti-cancer drug sensitivity of GICs in early phase of the differentiation. TMZ is widely used as an alkylating reagent in the treatment of high-grade glioma. We examined whether TMZ is effective on the viability during the differentiation of GICs, and how it effects to the differentiating GICs in the presence of RGD peptide. In GIC spheres, treatments of TMZ or combination of both TMZ and RGD had little effects on the cell viability and morphology (**Fig. 5B, C, D** **, left**). On the other hand, in differentiating GICs, TMZ treatment effectively inhibited the cell viability with dose-dependent manner (**Fig. 5B** **right**). Interestingly, these cellular effects of TMZ were further increased by the presence of RGD peptide (**Fig. 5B, C, D** **right**). To determine whether those cellular effects were caused by apoptotic events, GICs were analyzed by dual nuclear staining methods. In GIC spheres, treatments of TMZ, RGD peptide or combination of both treatments had a small effect on the cell phenotype. In contrast, in differentiating GICs, both of TMZ and RGD peptide effectively induced cell damages by increasing number of apoptotic cells. During the early differentiation phase (4 days) of GICs after serum induction, the combination treatment of TMZ and RGD peptide increased their apoptosis more than 1.7-fold compared with that of TMZ and control peptide (**Fig. 5C, D**), suggesting that the combination treatment of TMZ and integrin inhibitor RGD effectively increase the chemo-sensitivity of GICs during the initiation stage of differentiation. After the serum stimulation, GICs increases ECM and integrin inductions and form a microenvironment for further process, however, the specific inhibition of integrin on the differentiating GICs increases their sensitivity for anti-cancer drug, and this may suppress their further glioma propagation. We have summarized our hypothesis for the differentiation mechanism of GICs in the specific microenvironment, the so called “differentiation niche”, as a target for glioma chemotherapy, in **Fig. S6**.

**Figure 6:**
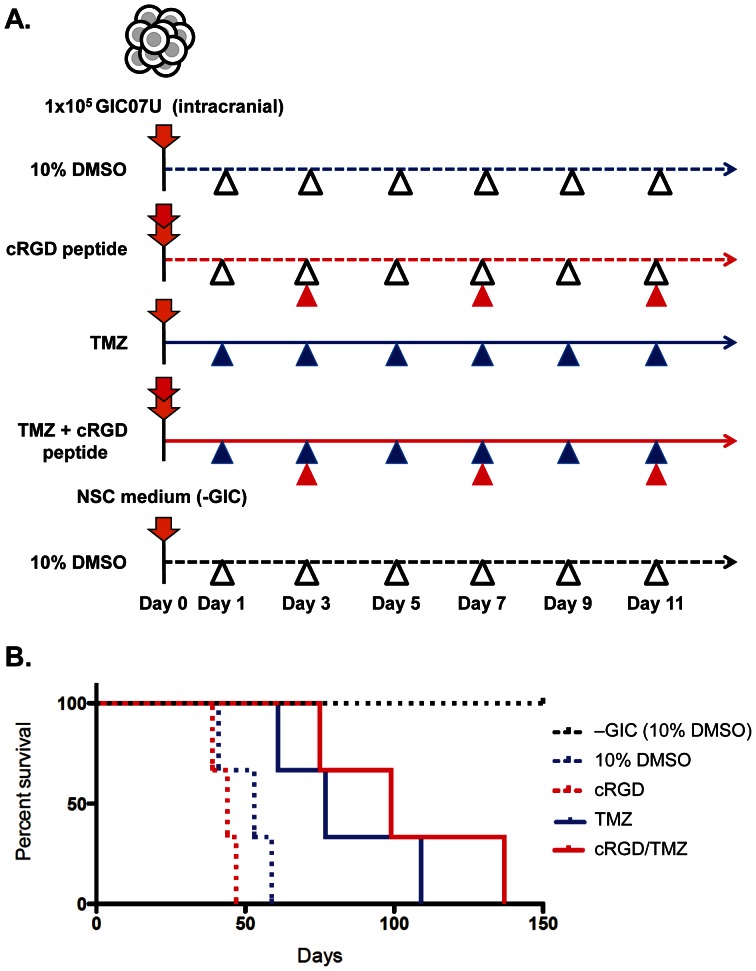
Combined treatment of cRGD peptide and TMZ prolonged mouse survival after GICs brain transplantation. ***A***. Therapeutic schedule for targeting GIC in the brain xenograft mouse model. Before transplantation of GIC, cells were pretreated with cRGD (100 µg/1×10^5^ cells) (red down arrow) or/and control medium (orange down arrow) for 30 min at 37°C, and subjected to the intracranial injection. After the GIC injection, cRGD (5 mg/kg mouse, red arrowhead), TMZ (7.5 mg/kg mouse, blue arrowhead), both of cRGD and TMZ, or control 10% DMSO (5 ml/kg mouse, white arrowhead) was injected intraperitoneally (i.p.) each time point according to the experimental design. The schedule of the i.p. administration was shown, control DMSO (10%): blue dotted line; cRGD peptide: red dotted line; TMZ: blue solid line; cRGD and TMZ: red solid line, for xenograft models, and 10% DMSO: black dotted line for control mice. ***B***. Kaplan-Meier survival curve of mice after GIC transplantation. The mice survivals after treatments of control 10% DMSO (blue dotted line, n = 3; median survival = 53 days), cRGD (red dotted line, n = 3; median survival = 44 days). TMZ (blue line, n = 3; median survival = 77 days), cRGD and TMZ combination (red line, n = 3; median survival = 100 days) were shown. All of control mice after NSC medium injection to brain without GIC (black dotted line, n = 3) were alive more than 5 months. *p*-value was determined with long-rank test (**p<0.0031).

To confirm our hypothesis *in vivo*, we finally analyzed the therapeutic effects of RGD and TMZ on the survival of mouse GIC xenograft model. After the intracranial transplantation of GICs (1×10^5^ cells) treated with or without cRGD peptide (cRGD), mice were further injected cRGD, TMZ, or both of them intraperitoneally every 2 to 4 days during the first 11 days as scheduled (**Fig. 6A**), and monitored daily for signs of morbidity up to 150 days. Kaplan-Meier survival curves of mice after the transplantation showed that the administration of cRGD only was not effective to their survivals (median survival = 44 days) compared with that of control DMSO (10%) (median survival = 53 days), however, the combination treatment of cRGD with TMZ had significantly prolonged the mice survival (median survival = 100 days), and this effect was more than those of TMZ treatment only (median survival = 77 days) (**Fig. 6B**). Autopsy of all mice brain tissues, performed immediately in each case, clearly showed that all of mice were dead of glioblastoma (data not shown). These results demonstrate that the early administration of cRGD with TMZ is highly effective to suppress the GIC derived glioblastoma progression and has an advantage for the survival in mouse GIC intracranial xenograft model. These results *in vivo* agree with the findings in the cellular experiments *in vitr*o, and support our hypothesis demonstrated in **Fig. S6**.

## Discussion

In this study, we established GIC clones whose xenografts developed into malignant gliomas in the mouse brain. Using these clones, we used integrated proteomics to identify molecules whose expression changes dynamically during the differentiation of GICs into glioma cells. In this analysis, GO terms such as ECMs, adhesion/cell communication, and membranes were present significantly among the gene/proteins upregulated during GIC differentiation. Among them, we focused on integrin family members and ECMs, such as COL4, LAMA2 and FN, which were commonly upregulated in all of the differentiating GIC clones at both mRNA and protein levels. In this paper, we demonstrate for the first time that the induction and interaction of integrin αV and FN, an ECM core protein, are key functions in the formation of a specific microenvironment, the so-called differentiation niche, by GICs, and that inhibitors such as blocking antibodies and RGD peptides coupled with anti-cancer drug might be useful for the abrogation of early events in GIC differentiation/proliferation and further glioma formation or propagation.

Quantitative proteome/transcriptome analyses are powerful tools for the characterization of biological phenomena and enable a detailed analysis of the chronological changes in mRNA and protein expression during differentiation. Although each of the techniques employed can be considered complementary, integration of the results of various studies is difficult due to differences in the quantification algorithms and language used in each study. In this study, we combined the data from DNA microarray and iTRAQ analyses by utilizing sequentially data mining software, such as an integrated gene/proteomic expression analysis chart application (iPEACH), Gene Springs, Subio platform, and KeyMolnet. By utilizing this strategy, we successfully identified functional protein groups to be focused, such as members of the ECMs and adhesion protein families as well as glioma markers such as MAPKs, which are associated with GIC differentiation.

ECMs are known to play a pivotal role in the development of tumors through their capacity to affect cellular migration and differentiation. Potential roles for ECMs in the stem cell niche and tumor microenvironment have been reported [12]. For example, COL4 significantly enhances the migration and differentiation of embryonic stem cells (ESCs) [13]. LAM is the most effective ECM for the maintenance of NSCs and GICs via its interaction with integrin α6 [2], [14]. Importantly, FN is a key regulator of the pre-metastatic niche involved in tumor formation [15], and a promoter of the migration of glioma cells [16]. However, ECM functions concerning on the GIC differentiation mechanism has not been clarified in detail. In this study, collagen family members, such as types XI, V, VI, IV, XVIII, V, I, XXIII, XXI, and XX, and laminin α2, β1, and β3, were shown to be increased during GIC differentiation, at mRNA or protein levels (**Table 1**). Upregulation of FN1 mRNA was significantly identified and demonstrated with immunocytochemistry and western blotting (**Fig. 4B** **and S2**) as well as immunohistochemistry (**Fig. 4A**), however, we could not detect it in the proteomics, suggesting that intracellular FN protein level is relatively lower than other ECMs because it will be dominantly secreted into the extracellular space and could not be collected in the cellular protein preparation for the proteomic analysis of GICs. Vitronectin is also known to be induced in glioma cells, which enhanced the cell migration [17]. However, in our study, neither mRNA nor protein expression of vitronectin was identified significantly. Although we focused only on COL4, LAMA2, and FN in this study because of their higher involvement in GIC differentiation, other ECMs may also contribute to the formation of the “differentiation niche”.

FN interactions to integrin families are involved in the differentiation of several types of stem cells [18] and/or in tumor metastasis/invasion including gliomas [16], however, their involvement in the promotion of GIC differentiation that leads to malignant glioma has not been reported so far. In this study, we demonstrated that GICs secretes FN, as an important core ECMs expressed in differentiating GICs, and that both FN/ECMs and serum factors are essential for GIC differentiation, since the expression of a differentiation marker GFAP in GICs was not stimulated by FN/ECMs in the absence of serum (**Fig. 3C**). The identity of the regulatory serum factor that directly induces the upregulation of ECMs/integrins for GIC differentiation is still unclear. Serum FN is unlikely to be involved in this induction because the addition of soluble FN without serum factors had a negative effect on GIC differentiation (data not shown).

Integrin family proteins are multifunctional cell adhesion molecules composed of α and β chains, which combine to form a wide variety of heterodimers [19], and are involved in stem cell regulation and/or cancer malignancy via their interactions with ECM proteins [18], [19]. We found that the expression of integrin α2, α5 and αV mainly increased during GIC differentiation. Other integrin family members, such as integrin α3, α4, α6, β1, β2 and β8, also showed increased expression at the mRNA level but not significant at the protein level (**Table 1**). Integrin αV forms heterodimers with integrin β1, β3, β5, β6 and β8 subunits [19]. Despite the large number of reports linking integrin αV to NSC differentiation or glioma malignancy [20], [21], the role of integrin αV and its associated β subunits in GIC adhesion/migration and differentiation remains to be defined. Our integrated proteomics showed that the protein expression of any integrin β subunits was not significant in differentiated GICs, suggesting that the heterodimers with multiple integrin β subunits may support GIC differentiation via the RGD-binding motif. Integrin α2 is expressed in ESCs and NSCs [22], and it stimulates cell adhesion/migration [13]. Integrin α5 is also expressed on NSCs [22] and human gliomas [23]. In this study, the expression of integrin α2 and α5 during GIC differentiation was found to be increased; however, antibodies directed against them did not inhibit GIC differentiation, suggesting that integrin α2 and α5 may be a supportive integrin for GIC adhesion/migration on ECMs and not a main factor involved in the early stage of GIC differentiation.

TMZ has been currently used for the initial chemotherapeutic agent for GBM. However, despite aggressive treatment including surgery, adjuvant TMZ-based chemotherapy, and radiation therapy, GBM still has a poor prognosis and the median survival is less than 15 months from diagnosis [24]. Recently, an integrin αV antagonist, a cyclic RGD-containing peptide termed cilengitide, was reported to improve the prognosis of patients with glioma by increasing cell apoptosis and inhibiting glioma invasion and proliferation [20], [21], [25]. In this study, we demonstrate that inhibitors of the integrin αV and ECM interaction, such as RGD peptide or blocking antibodies, coupled with TMZ treatments could be effective as early stage therapeutics against glioma recurrences. In fact, our animal studies clearly show that the proliferation and differentiation of GICs immediately after the intracranial transplantation, which may mimic the early stage of postoperation for malignant gliomas, can be effectively inhibited by the combination therapy with integrin inhibitor cRGD and TMZ.

In addition, it was also suggested that integrin inhibition coupled with the methylation of a DNA repair enzyme O-6-methylguanine-DNA methyltransferase (MGMT) gene promoter could be involved in the increased TMZ sensitivity of gliomas [20], [21]. MGMT is known to antagonize the genotoxic effects of alkylating agents such as TMZ. In our integrated proteomics, downregulation of MGMT mRNA were observed in the differentiating GICs (data not shown), therefore, increased TMZ sensitivity during GIC differentiation may partly be caused by MGMT downregulation. The study for this issue is under our investigation. The mechanism of the effect of the RGD-containing peptide on the glioma cells may be more understandable when we postulate that the differentiating GICs in the differentiation niche (**Fig. S6**), just after the differentiation switch being on, could have the highest potentiality as the target for RGD and TMZ, because GICs in the stem cell niche is less sensitive against the RGD as well as TMZ.

Here, we firstly demonstrate that the RGD related peptide or integrin αV inhibitor is a strong suppressor for early event of GIC differentiation and proliferation, and a combination of RGD treatment with anti-cancer drug TMZ could have the highest inhibitory potential against the glioma recurrence that may be regulated by the GICs in the differentiation niche.

## Materials and Methods

### Ethics statement

All human brain tumors were obtained at Kumamoto University Hospital after obtaining written informed consent and the approval of the Institutional Review Board of Kumamoto University, and the Ministry of Health and Welfare of Japan (Genome 110, 2008, revised in 2010). The individuals in this manuscript have given written informed consent in the Kumamoto University Consent Form as outlined Genome 110, 2008. Mouse studies were conducted in accordance with the guidelines for animal protection used for experimental purposes in Japan. All animal experiments were approved and permitted by the Animal Care and Research Advisory Committee of Kumamoto University (Permit Number: D23-215). All surgery was performed under sodium pentobarbital anesthesia, and all efforts were made to minimize suffering. All of the mice were monitored daily for signs of morbidity. Survival time reflects the time required for the animals to reach the following parameters, rigid paralysis or minimal joint movement, foot not being used for forward motion, the inability of a mouse to right itself within 30 sec of being placed on a side, rapid weight loss exceeding 15%. The animal weight was measured at day 0 and remeasured every 2 days until the end of the experiment. Once mice reached their end point, they were euthanized via cervical dislocation and the date and cause of their death was recorded. We have strived to replace the use of animals in our studies with in vitro or non-invasive assays, reduce the number of animals utilized, and refined our use of animals to minimize their suffering and maximize the data extracted from each experiment.

### Establishment of GICs and cell culture

Human brain tumors from four GBM and one Anaplastic Oligodendroglioma (AO) patients were obtained at Kumamoto University Hospital. Within 4 hours of tumor removal, tissues were subjected to GIC preparation. The tissue cells were disassociated, separated from cell debris, and cultured in NSC medium; Neurobasal-A Medium (GIBCO/Invitrogen), B-27 (1∶50; GIBCO/Invitrogen), heparin (5 µg/ml; SIGMA), and GlutaMax-1 (GIBCO) containing recombinant hFGF-2 (20 ng/ml; PeproTech Inc), recombinant hEGF (20 ng/ml; PeproTech Inc), recombinant hLIF (20 ng/ml; Chemicon Inc), and insulin (10 ng/ml, SIGMA). The GIC spheres observed after 4–5 weeks cultivation were treated with Accumax (Innovative Cell Technologies). After successive cloning, each cloned cells (1×10^6^ cell/ml) were sub-cultured, once a week, and the clones that could form spheres within a few days were subjected to the serial passages for more than 1–2 years.

### Integrated proteome analysis

For transcriptomics, total RNA from GIC03A and GIC03U clones on day 2 or 7 of culture in NSC medium with or without 10% FCS was subjected to the analysis with Affymetrix human gene U133 Plus 2.0 array according to the manufacturer's protocol. The raw data are available through the National Center for Biotechnology Information's Gene Expression Omnibus (GEO series accession number: GSE43762). Simultaneously, the proteins extracted from the same set of cells fractionated to 44 fractions by a cation exchange column (Mono S column, GE Healthcare) were subjected to LC-shot gun analyses using the 8-plex iTRAQ method as described previously [7], running parallel uses of three different kinds of tandem MS systems; nanoLC-ESI (QSTAR-Elite, and TT-5600) and MALDI (TOF/TOF-5800) (AB Sciex) (total 132 run:44 fraction ×3 with 8-plex data). Protein ratios are given as averages from the three iTRAQ data obtained from the three tandem MS analyses (described above) of 2 sets of GICs treated with (condition of the differentiation) or without (condition of the sphere formation) serum for 2 days or 7 days. A confidence cutoff for protein identification >95% was applied. The intensity of 113, 114, 115, 116, 117, 118, 119, and 121 atomic mass unit signature mass tags generated upon MS/MS fragmentation from the iTRAQ-labeled tryptic peptides were used to quantify the relative level of peptides and hence proteins in each sample. To determine the relative abundance of proteins in GIC differentiation at 2 days, or at 7 days versus GIC sphere at 2 days, peptide and protein ratio were expressed using the 113 or 117 tag to label GIC03A or 03U sphere at 2 days as the denominator, respectively. Total data from DNA array and iTRAQ were combined by integrated gene/proteomics expression analysis chart method (iPEACH; PCT/JP2011/58366) [26] and Subio Platform (Subio), extracted and subjected to statistical and GO analyses by GeneSprings (Agilent) and KeyMolnet (IMMD).

### Mouse transplantation and in vivo chemical administration

The experiments were performed according to the guidelines of the Animal Experimental Committee of Kumamoto University. 1×10^3^ [GIC03A (n = 6), 03U (n = 6)] or 1×10^5^ cells [GIC03A (n = 6), 03U (n = 6) and 07U (n = 3)] were injected into the brains (subventricular area) of 5–8 week-old female NOD/SCID mice, which were monitored daily for signs of morbidity. For *in vivo* chemical administration, cyclic RGD (cRGD, Peptide Institute. Inc) and temozolomide (TMZ, Sigma) solutions were made fresh each day in distilled H_2_O and 10% DMSO at a concentration of 1 mg/ml and 1.5 mg/ml, respectively. Before intracranial transplantation of GICs, cells for the cRGD administration group were pretreated with cRGD (100 µg/10^5^ cells) for 30 min at RT and subjected to the injection. After the GIC injection, cRGD (5 mg/kg mouse), TMZ (7.5 mg/kg mouse), combination of cRGD and TMZ (5 mg and 7.5 mg/kg mouse, respectively), or control 10% DMSO (5 ml/kg mouse) were injected intraperitoneally each time according to the experimental design. All surgery was performed under sodium pentobarbital anesthesia, and all efforts were made to minimize suffering.

### Immuno-cytological/histological chemistry

For immunocytochemistry, cells were fixed with 4% paraformaldehyde, permeabilized, and blocked with 3% BSA, and reacted with the primary antibodies: rabbit polyclonal anti-CD133, -COL4, rat polyclonal anti-LAMA2, mouse monoclonal anti-integrin α2 (Abcam), rat polyclonal anti-CD44, mouse monoclonal anti-FN, anti-integrin αV (BD Bioscience), -Sox2 (R&D System), -class III-β-tubulin (Covance), and -GFAP antibody (Millipore). Cells were washed with PBS and incubated with the appropriate secondary antibody: goat anti-mouse Alexa 488 IgG or goat anti-rabbit Alexa 546 IgG (Invitrogen). Nuclei were counterstained with 4′,6-diamidino-2-phenylindole, dilactate (DAPI) (Molecular Probes). For the immunohistochemistry, the paraffin sections of tumors pretreated were neutralized with 3% H_2_O_2_ in methanol after 30 sec antigen retrieval in citrate buffer (Nichirei). Sections were blocked with 5% goat serum (DAKO) and then treated overnight at 4°C with anti-human Ki-67 mouse antibody (DAKO) to estimate the proliferative index, or with the same set of antibodies described above. After treatment with peroxidase labeled secondary antibody (Histofine Simple Stain MAX-PO), color reactions were performed with a DAB substrate kit.

### Western blot analysis

GIC lysates were subjected to SDS-PAGE, followed by the immunoblot analysis, using the same sets of antibodies used for immunocytochemistry as described. The fluorescence or ECL-chemiluminescence of secondary Cy5-anti-mouse IgG, Cy2-anti-rabbit IgG, or HRP-anti-Rat IgG antibodies were analyzed using Typhoon 9400 (GE Healthcare) or LAS 4000 mini (Fujifilm), respectively.

### GIC spheres differentiation assay

GIC spheres were plated onto NSC medium containing 10% FCS in non-coated, or COL4-, LAM- (BD Bioscience), FN- (Biomedical Technologies) and poly-L-lysine (PLL)-coated dishes and cultured for 2–7 days. In the inhibition experiment, GIC spheres were pre-treated with blocking antibodies against integrin α2 (Abcam), αV, α6 (Biolegend), α5, β1, and control IgG1 (R&D), or with the binding peptide, GRGDTP, GRGESP, and DGEA (Anaspec), for 30 min before seeding on each dish. Cellular morphological changes were monitored on a time-lapse microscope IX81 equipped with a CO_2_ incubation chamber system (OLYMPUS) using MetaMorph software Ver. 7.5.5.0 (Molecular Devices).

### Cell proliferation assay

GICs after the dissociation with Accumax were seeded in 96-well plates (2×10^3^ cells/well). After the treatment with the binding peptides, GRGDTP, GRGESP, or DGEA for 30 min, cells were incubated with NSC medium with or without 10% FCS, in the presence or absence of TMZ. Each well of cells was counted by use of Cell Counting kit-8 (WST-8, Dojindo) according to manufacture recommendation. Eight replicate wells were used for each condition.

### Apoptosis assay

GICs were dissociated with Accumax, and seeded on 24-well plates (1×10^4^ cells/well). After the treatment with the peptides for 30 min, cells were incubated with NSC medium with or without 10% FCS. After 6 hours incubation, cells were then treated with 0–500 µM of TMZ as indicated. Every after 24 hours incubations, the cells were fed NSC medium with peptides. After 4 days cultivation, cells were stained with Propidium Iodide (PI) (2 µg/ml; Sigma) for counting the apoptotic cells, and Hoechst 33342 (2 µg/ml; Molecular Probes) for staining the total cells [7]. The PI and Hoechst stained cells (3,000–7,000 cells/one field) were counted in six fields in duplicated tests and the identical independent experiments were repeated twice. The cell counting was performed by MetaMorph software ver. 7.5.5.0, using cell counter mode (Molecular Device).

### Quantitation and statistical analysis

Relative quantitation and statistics for DNA array and iTRAQ analyses were mainly performed by GeneSprings (Agilent) and ProteinPilot (AB Sciex), respectively. For quantitative western blotting, fluorescence/chemiluminescence intensities labeled on the each protein were visualized using a Typhoon 9400 imager, and quantified with ImageQuant TL software (GE Healthcare) using actin as an internal control. All data were presented as mean +S.E. of more than three independent experiments run in duplicate. *p*-values were determined by Student's t-test. *p*<0.05 was considered significant.

## Supporting Information

Figure S1 **Establishment and characterization of GICs.**
(TIF)

Figure S2 **Validation of proteins upregulated in differentiating GICs.**
(TIF)

Figure S3 **Effects of blocking antibodies against integrin α5, α6 and β1 during GIC differentiations.**
(TIF)

Figure S4 **Upregulation of MAPK signaling in differentiating GICs.**
(TIF)

Figure S5 **Inhibition of MAPK signaling in differentiating GICs by RGD peptide.**
(TIF)

Figure S6 **The postulated regulation mechanism of GICs as a therapeutic target in the differentiation niche.**
(TIF)

Movie S1 **Time-lapse image of serum stimulated GIC spheres in the presence of IgG control.**
(MP4)

Movie S2 **Time-lapse image of serum stimulated GIC spheres in the presence of anti integrin α2 antibody.**
(MP4)

Movie S3 **Time-lapse image of serum stimulated GIC spheres in the presence of anti integrin αV antibody.**
(MP4)

Movie S4 **Time-lapse image of serum stimulated GIC spheres in the presence of GRGESP peptide.**
(MP4)

Movie S5 **Time-lapse image of serum stimulated GIC spheres in the presence of DGEA peptide.**
(MP4)

Movie S6 **Time-lapse image of serum stimulated GIC spheres in the presence of GRGDTP peptide.**
(MP4)

Table S1 **Overrepresented biological process and cellular component by enrichment analysis of mRNA and Proteins differentially expressed in response to serum stimulation.**
(TIF)

Table S2 **Heatmap visualization of glioma markers upregulated in differentiating GICs.**
(TIF)
