# Supplementary figures and images for: Glioma Initiating Cells Form a Differentiation Niche Via the Induction of Extracellular Matrices and Integrin αV
Source: PLoS One. 2013 May 21;8(5):e59558. doi: 10.1371/journal.pone.0059558 (PMC3660593; doi:10.1371/journal.pone.0059558)

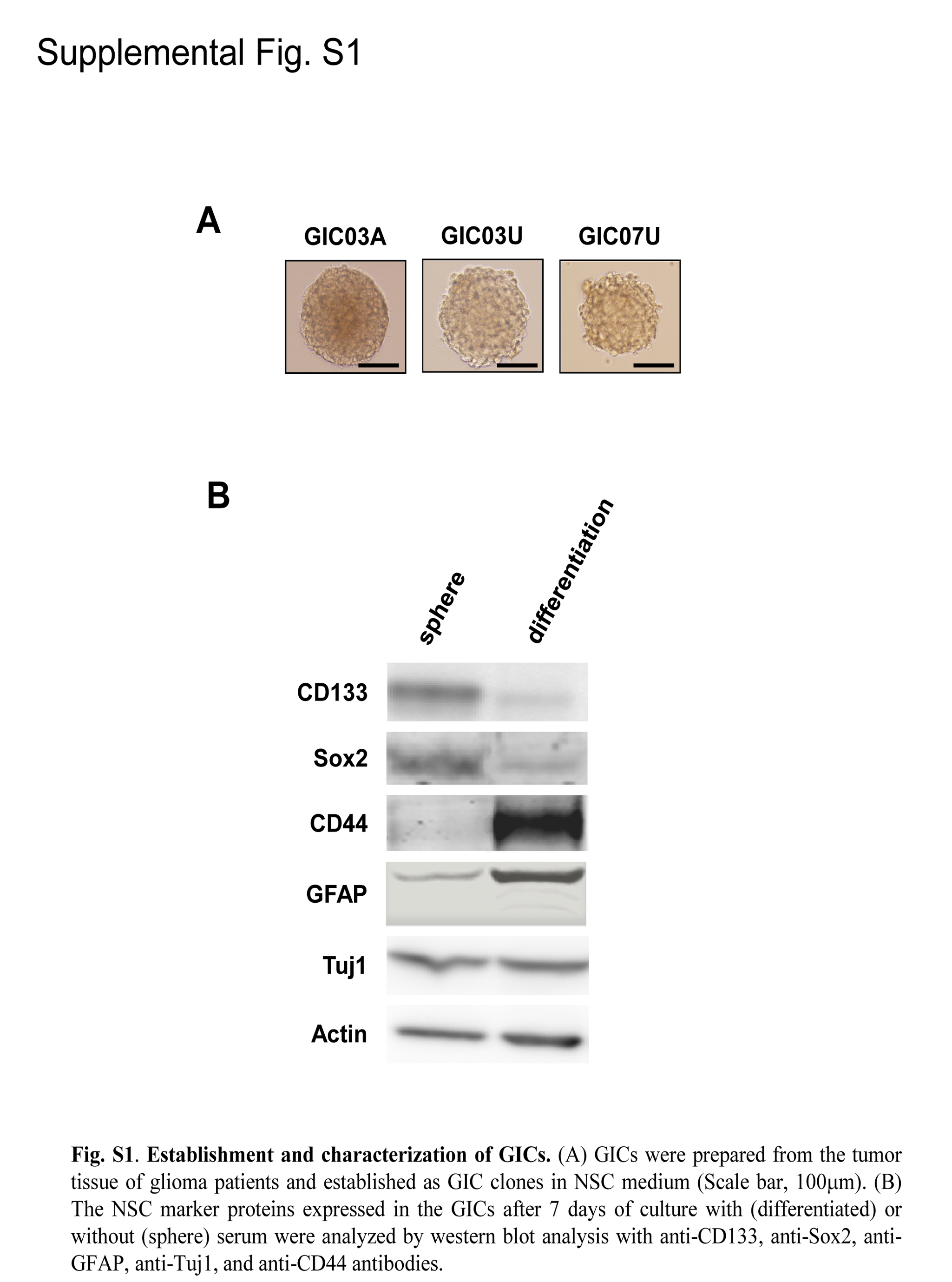

Supplement: Figure S1 — Establishment and characterization of GICs. (TIF) [file pone.0059558.s001.tif]

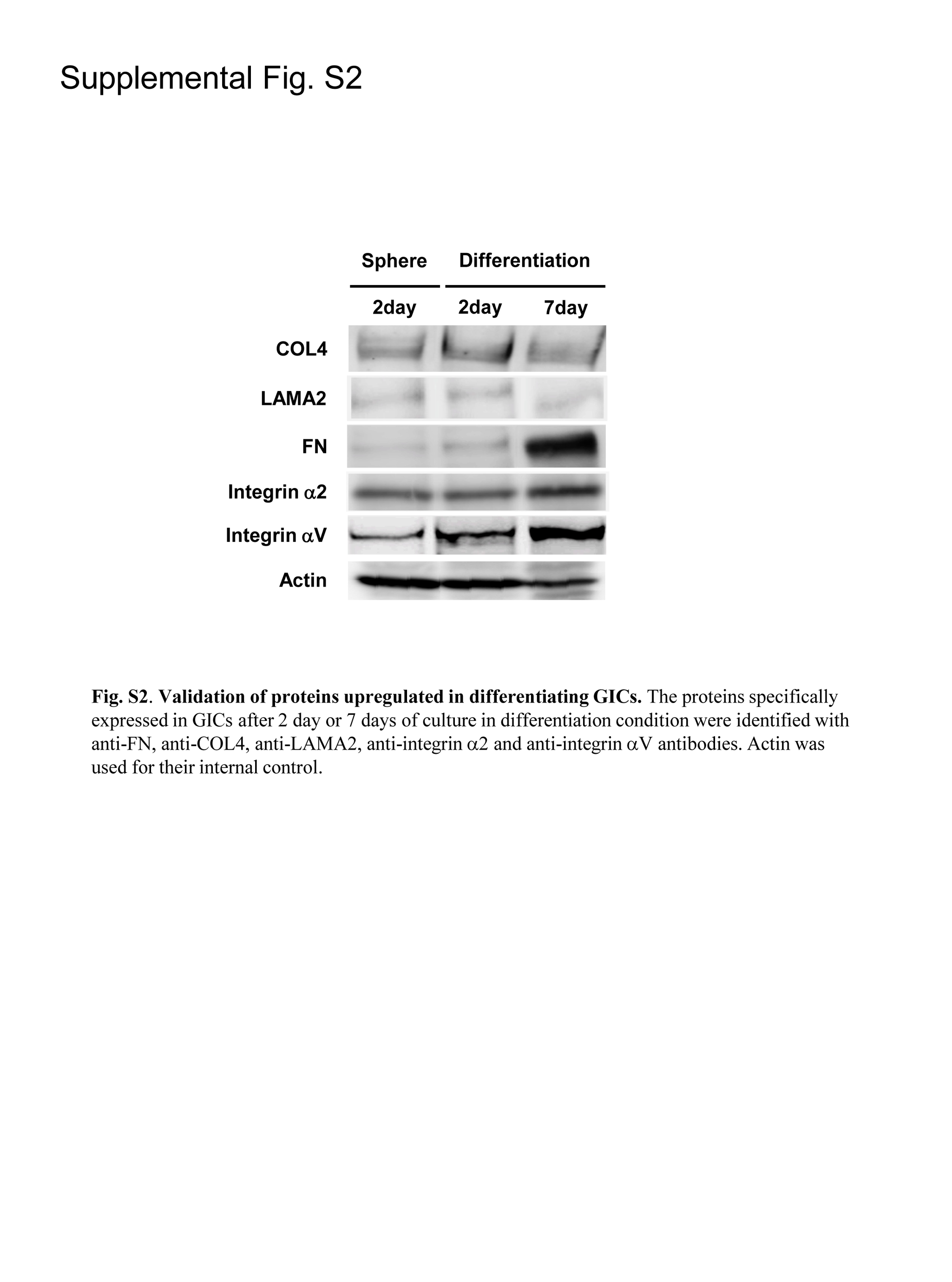

Supplement: Figure S2 — Validation of proteins upregulated in differentiating GICs. (TIF) [file pone.0059558.s002.tif]

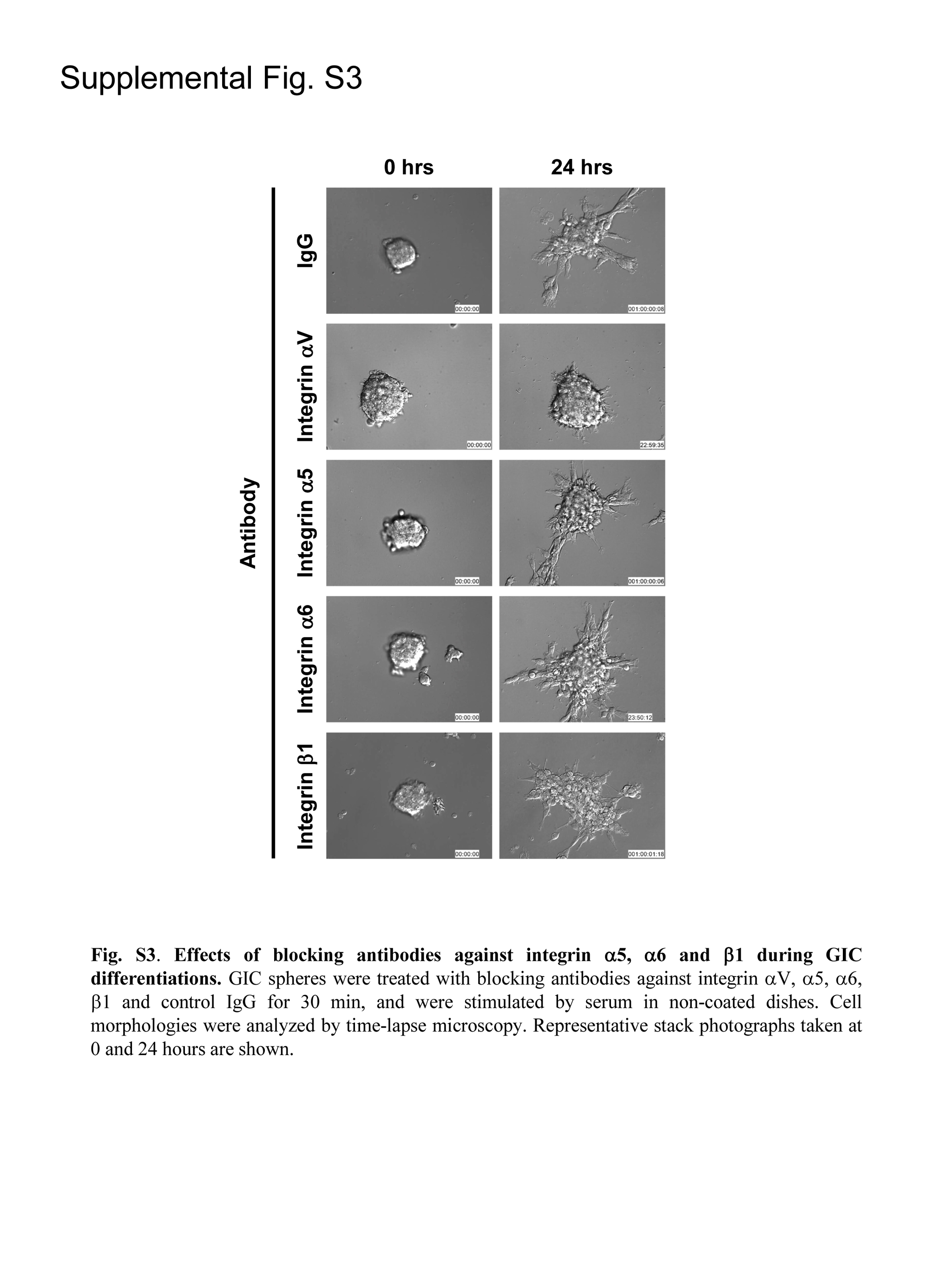

Supplement: Figure S3 — Effects of blocking antibodies against integrin α5, α6 and β1 during GIC differentiations. (TIF) [file pone.0059558.s003.tif]

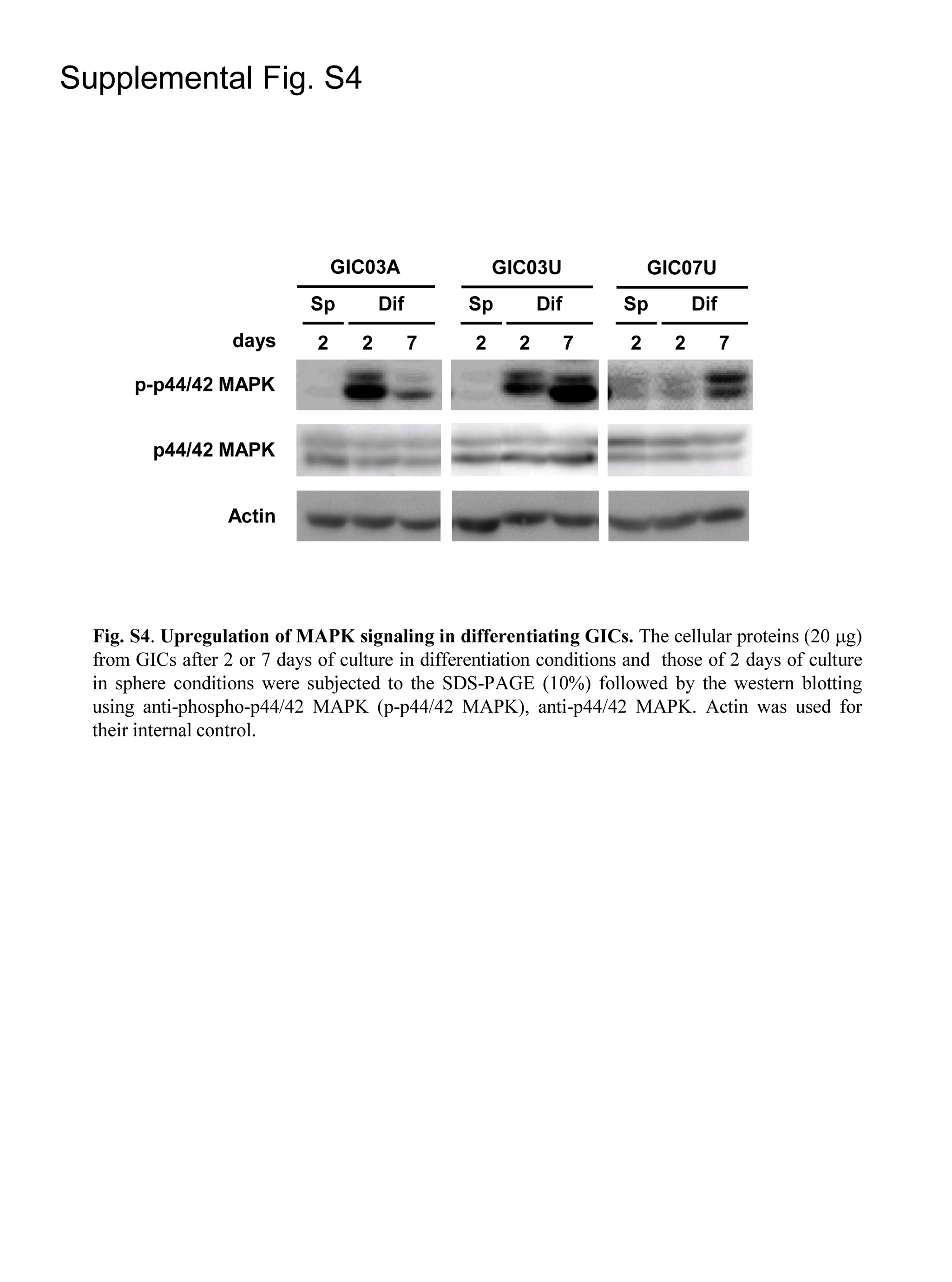

Supplement: Figure S4 — Upregulation of MAPK signaling in differentiating GICs. (TIF) [file pone.0059558.s004.tif]

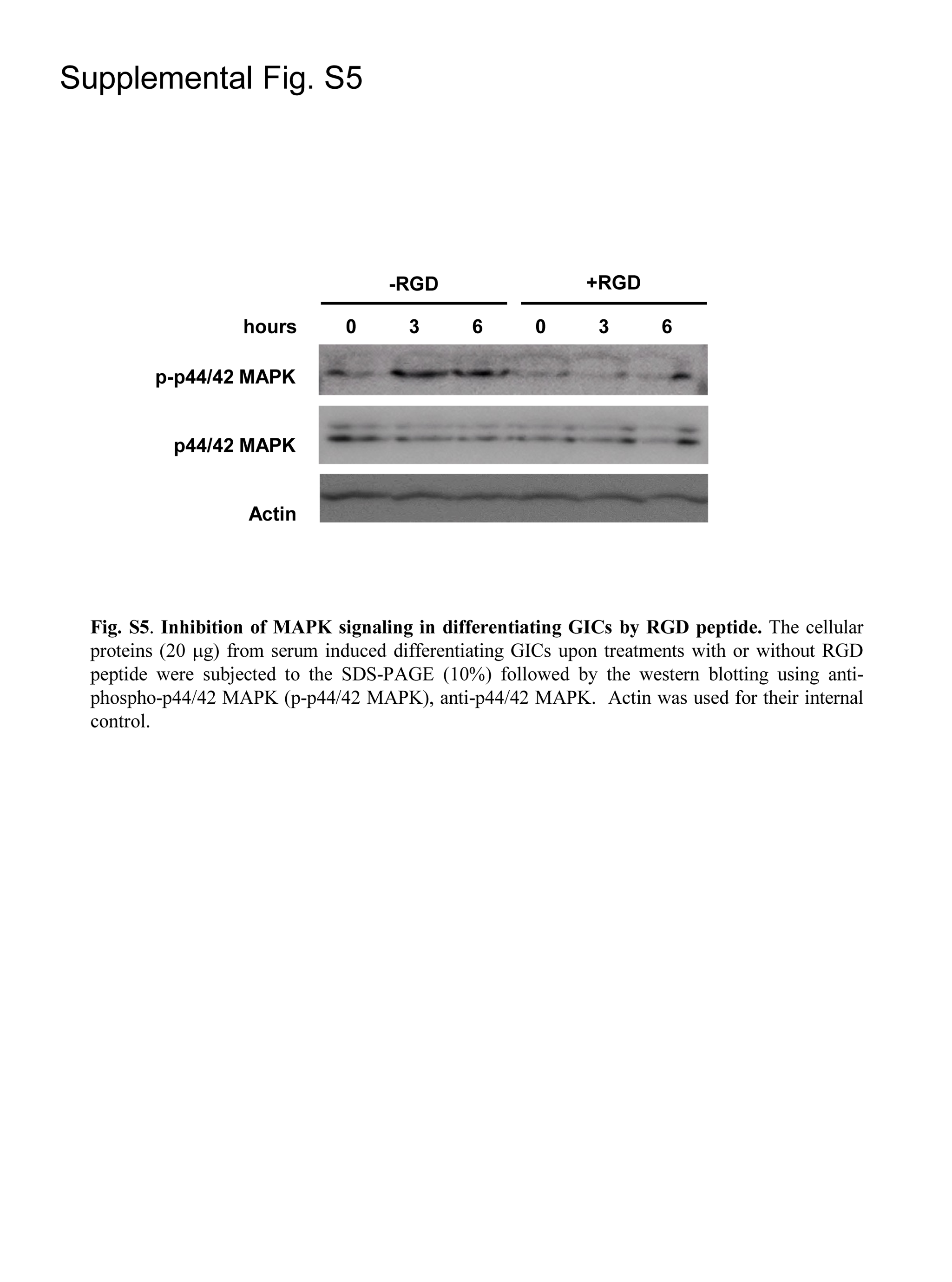

Supplement: Figure S5 — Inhibition of MAPK signaling in differentiating GICs by RGD peptide. (TIF) [file pone.0059558.s005.tif]

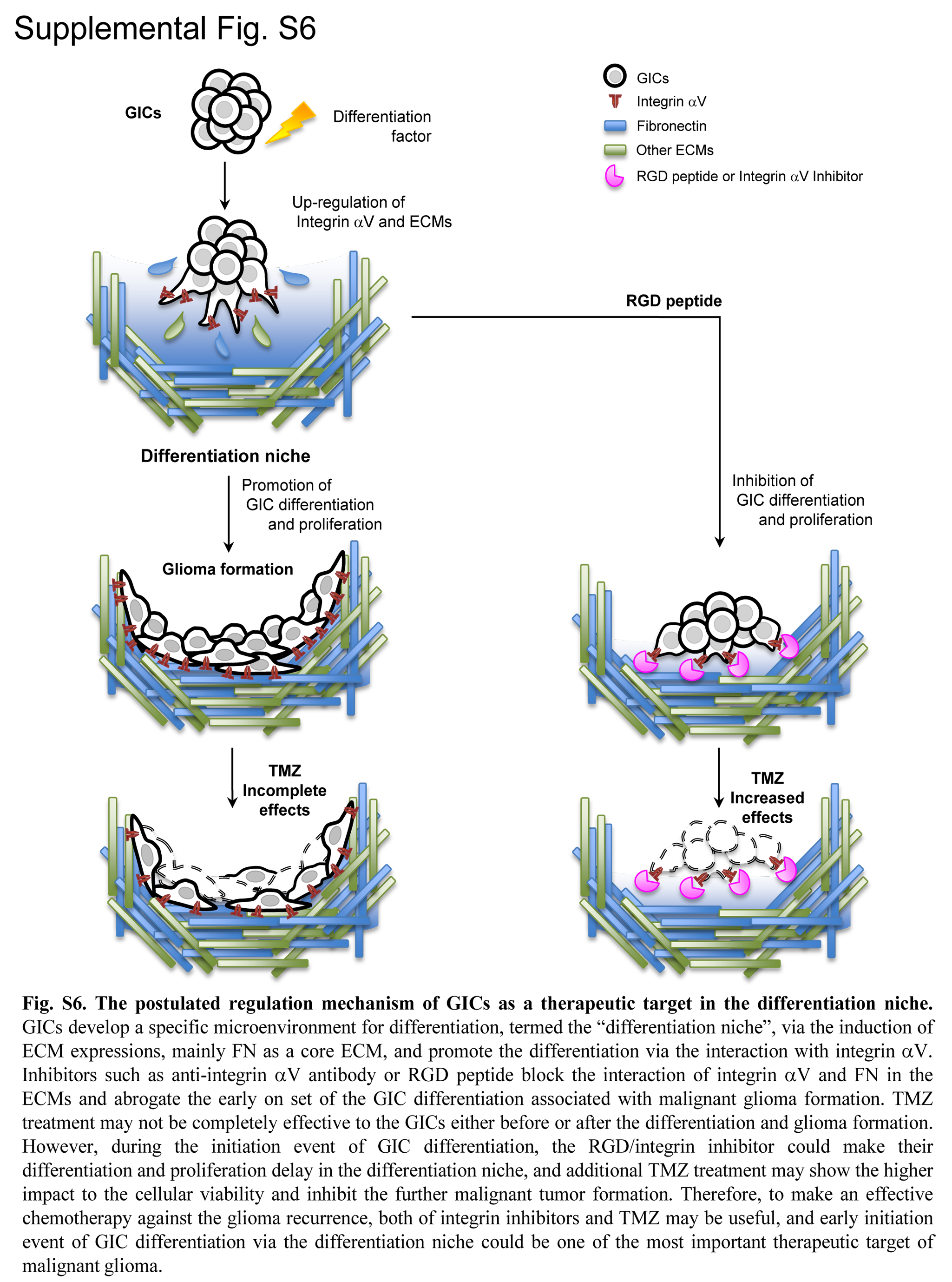

Supplement: Figure S6 — The postulated regulation mechanism of GICs as a therapeutic target in the differentiation niche. (TIF) [file pone.0059558.s006.tif]

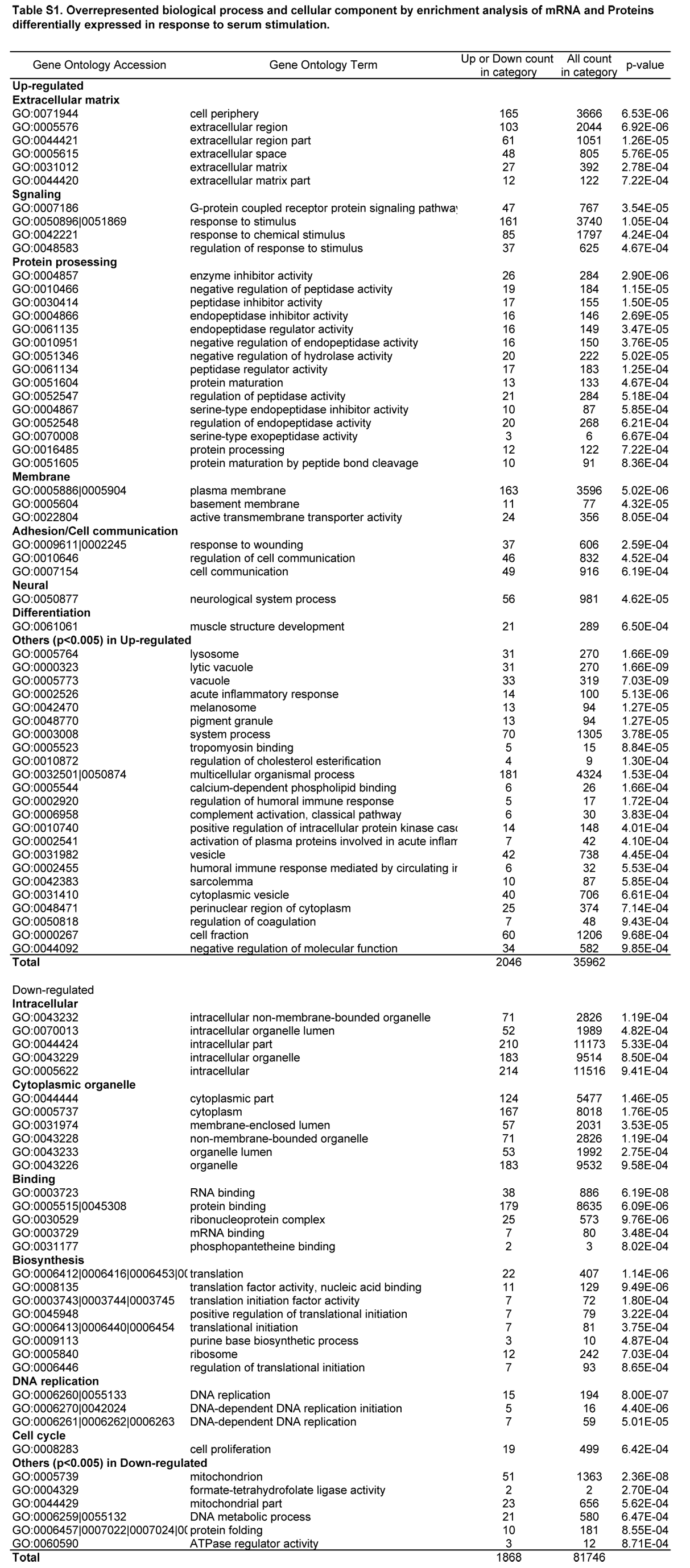

Supplement: Table S1 — Overrepresented biological process and cellular component by enrichment analysis of mRNA and Proteins differentially expressed in response to serum stimulation. (TIF) [file pone.0059558.s013.tif]

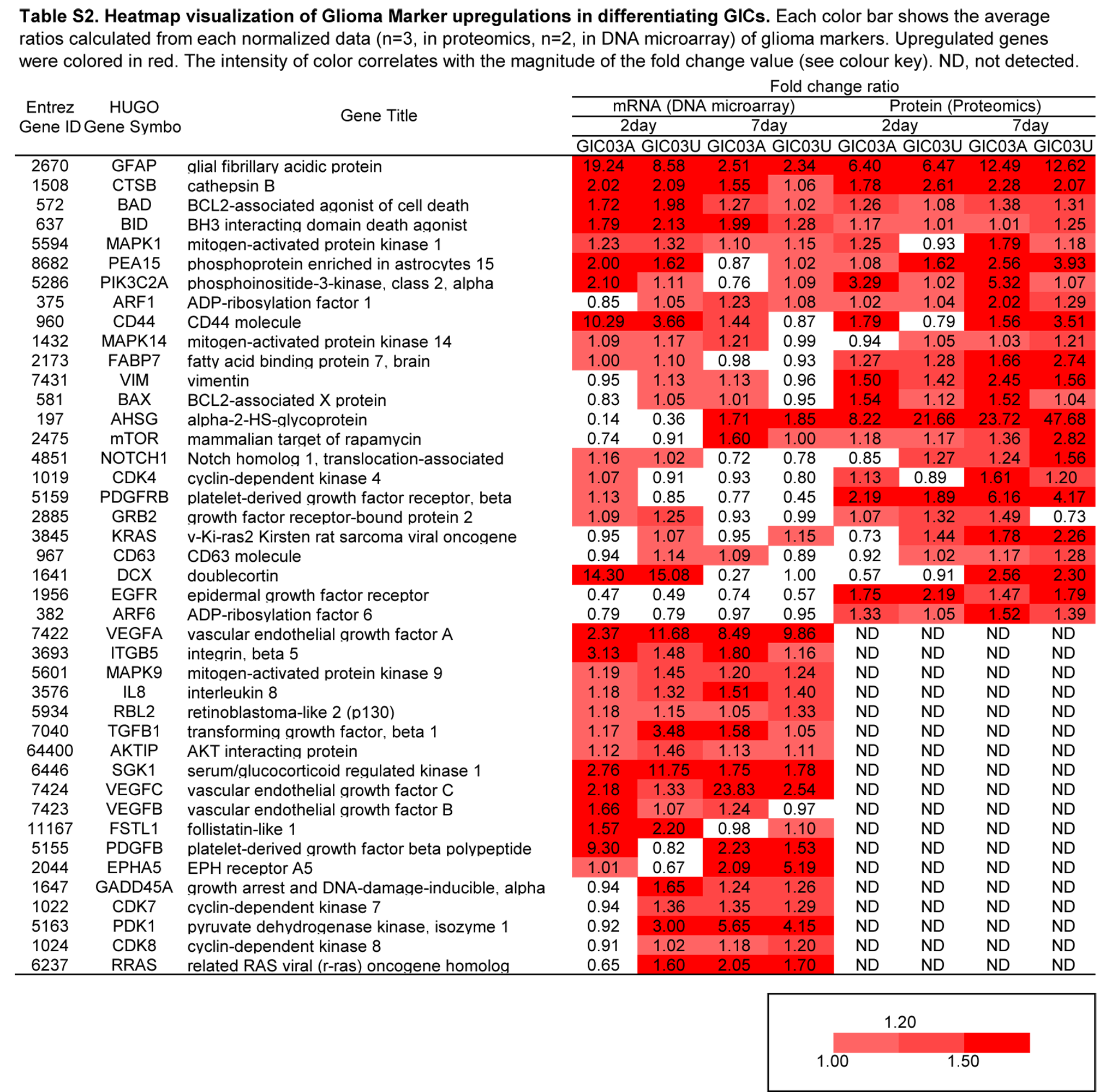

Supplement: Table S2 — Heatmap visualization of glioma markers upregulated in differentiating GICs. (TIF) [file pone.0059558.s014.tif]
